# Validation of risk prediction for outcomes of severe community-acquired pneumonia among under-five children in Amhara region, Northwest Ethiopia

**DOI:** 10.1371/journal.pone.0281209

**Published:** 2023-02-15

**Authors:** Zelalem Alamrew Anteneh, Hunegnaw Enyew Arega, Kebadnew Mulatu Mihretie

**Affiliations:** 1 Department of Epidemiology, School of Public Health, Bahir Dar University, Bahir Dar, Ethiopia; 2 Licha Health Center, East Estie, South Gondar Zone, Amhara Region, Ethiopia; World Health Organization, SWITZERLAND

## Abstract

**Background:**

Globally there are over 1,400 cases of pneumonia per 100,000 children every year, where children in South Asia and Sub-Saharan Africa are disproportionately affected. Some of the cases develop poor treatment outcome (treatment failure or antibiotic change or staying longer in the hospital or death), while others develop good outcome during interventions. Although clinical decision-making is a key aspect of the interventions, there are limited tools such as risk scores to assist the clinical judgment in low-income settings. This study aimed to validate a prediction model and develop risk scores for poor outcomes of severe community-acquired pneumonia (SCAP).

**Methods:**

A cohort study was conducted among 539 under-five children hospitalized with SCAP. Data analysis was done using R version 4.0.5 software. A multivariable analysis was done. We developed a simplified risk score to facilitate clinical utility. Model performance was evaluated using the area under the receiver operating characteristic curve (AUC) and calibration plot. Bootstrapping was used to validate all accuracy measures. A decision curve analysis was used to evaluate the clinical and public health utility of our model.

**Results:**

The incidence of poor outcomes of pneumonia was 151(28%) (95%CI: 24.2%-31.8%). Vaccination status, fever, pallor, unable to breastfeed, impaired consciousness, CBC abnormal, entered ICU, and vomiting remained in the reduced model. The AUC of the original model was 0.927, 95% (CI (0.90, 0.96), whereas the risk score model produced prediction accuracy of an AUC of 0.89 (95%CI: 0.853–0.922. Our decision curve analysis for the model provides a higher net benefit across ranges of threshold probabilities.

**Conclusions:**

Our model has excellent discrimination and calibration performance. Similarly, the risk score model has excellent discrimination and calibration ability with an insignificant loss of accuracy from the original. The models can have the potential to improve care and treatment outcomes in the clinical settings.

## Introduction

Pneumonia is a form of an acute respiratory tract infection that causes inflammation of the pulmonary parenchyma (alveoli and bronchioles). It is an acute and lower respiratory tract infection caused by a number of infectious agents, including viruses, bacteria, and fungi, and the most severe form of pneumonia is due to bacterial causes [1, 2]. For the purpose of an intervention, pneumonia is commonly classified into community acquire, hospital acquire, aspiration, and ventilator-associated pneumonia; it is also often classified based on the area of the lung it affects [3]. Pneumonia affects all age groups; however, children are severely and disproportionately affected than any other age group. This could be associated with younger people might have a weakened immunity due to malnutrition and other diseases [4, 5].

Community acquire pneumonia (CAP) in children can be caused by bacteria, viruses, or mixed infections. Pieces of evidence showed that the etiology of CAP in hospitalized children accounted for 33% viral, 19% bacterial, and 33% mixed infections [6, 7].

Pneumonia is the single largest infectious cause of death in children, in 2019 pneumonia claimed the lives of over 800,000 children less than 5 years of age, which implies nearly, 2,200 children die daily. This accounts for 14% of all deaths of children less than five years old but 22% of all deaths in children aged 1 to 5 [1, 8]. Globally, there are over 1,400 cases of pneumonia per 100,000 children, implying 1 pneumonia case per 71 children every year, where children in South Asia and Africa are disproportionately affected [9].

Evidence showed that the prognosis of pneumonia in children is good; most cases of viral pneumonia resolve without treatment; common bacterial pathogens and atypical organisms respond to antimicrobial therapy. Long-term alteration of pulmonary function is rare [10, 11]. Although the definition for severe community acquired pneumonia (SCAP) isn’t uniform; based on WHO guideline for pneumonia control strategy for resource limited settings; severe community acquired pneumonia is defined as children who had chest indrawing with or without fast breathing or children who had any general danger signs such as: inability to breast feed/eat, vomiting everything, grunting, lower chest endowing, altered mental status) that requires referral to the closest higher-level health facility for a high intensity monitoring and treatment [12–14].

A child diagnosed with severe community-acquired pneumonia (SCAP) can be treated with parenteral or oral roots of administration of medicines [15]. Those children diagnosed with SCAP should be treated with parenteral antibiotics at the health facility level [2, 16]. Although patients placed on a protocol-driven pneumonia clinical pathway are more likely to have favorable outcomes, there is a chance that the outcomes of the clinical care could be poor because of malnutrition, immunosuppression, prematurity, co-existing illness, and others [7, 17–19]. In light of the clinical care, it is very important to determine the prognosis and predict the outcomes of CAP to provide an individual case-based intervention for patients with higher risks of poor outcomes of SCAP. Therefore, we developed a prediction model and risk scores to assist the clinical judgment made by the health care workers.

## Methods and materials

An institutional based prospective cohort study was conducted among children less than 5 years of age hospitalized with severe community-acquired pneumonia in Amhara Regional State, Northwest Ethiopia. The study was conducted from February to May/ 2020 in public referral hospitals (Felege-Hiwot Comprehensive Specialized Hospital (FHCSH), Tibebe Ghion Specialized Hospital (TGSH), and Debre-Markos Comprehensive Specialized Hospital (DMCSH). FHCSH and TGSH are located in Bahir Dar city, the capital of Amhara Regional State at a distance of 565kms from Addis Ababa capital city of Ethiopia. Both are governmental Hospitals providing inpatient services for children in the pediatric ward and have an emergency outpatient department (EOPD) which can admit for 24-72hours. And DMCSH is Referral Hospital in East Gojam Zone in Debre Markos city. Debre Markos city is the capital of East Gojam located at a distance of 185kms away from Addis Ababa. Similarly, it is a governmental hospital providing both inpatient and outpatient health care services in the region.

The study domain for our study is children from 2–59 months of age diagnosed with severe community-acquired pneumonia based on World Health Organization diagnosing (WHO) criteria of Pneumonia Severity Index (PSI) at the selected hospitals admitted to pediatrics emergency outpatient department or pediatrics wards. In the hospitals, for the purpose of intervention and classifications of pneumonia, SCAP is defined as children who reported chest indrawing with or without fast breathing or children who had any general danger signs such as: inability to breast feed/eat, vomiting everything, grunting, lower chest endowing, altered mental status [13, 15]. Children diagnosed with SCAP but died before the start of medication, and children diagnosed with SCAP at admission, but later whose diagnosis changed at any time during the follow-up were excluded from our study.

The sample size was determined based on the minimum standard of 10 events per candidate predictor considered, thus the formula used was N = (n × 10)/I, where N is the sample size, n is the number of candidate predictor variables, and I is the estimated event rate in the population [20]. We have 18 potential prognostic determinants for severe community-acquired pneumonia, and the prevalence of one of the components for poor outcomes of severe community-acquired pneumonia was 35% [21]. Therefore, the calculated sample size was 514, and adding 10% refusal for participation rate, the total sample size required was 565 children. As a sampling procedure, we allocated the sample size to the three hospitals above based on their previous monthly admission rates taken from DHIS reports. So, 203 cases from FHCSH, 147 cases from TGSH, and 215 cases from DMCSH were selected consecutively.

### Outcome measurement

Poor outcomes of severe community-acquired pneumonia said to be occurred if treatment failure or antibiotic change or staying longer in the hospital or death occurred during follow-up days, otherwise, good outcome.

### Operational definitions for some terminologies

Treatment failure: treatment failure operationalized as persistence of features of severe pneumonia after initiation of antimicrobial therapy or worsening clinical condition within 48–72 hours of the commencement of the antibiotics [22, 23].

Antibiotic change: is a shift from one drug to the other (first line to second line) after taking at least two doses &, A longer stay in the hospital: staying for more than five days in the hospital regarded as longer [7].

Death: If a child has died during the hospitalization days and confirmed by health professionals.

Complete Blood Count (CBC)—It is a composite variable. Laboratory findings in an increased in the level of platelets, white bloods, neutrophils, or presence of anemia categorized (abnormal), otherwise, the finding is normal.

### Prognostic determinants

Sociodemographic factors (age, sex, immunization status, delayed presentation (> 5 days), exclusive breastfeeding, and complementary feeding), clinical at presentation (breathlessness, fever, pallor, cyanosis, grunting, and vomiting), co-morbidities (Human Immune Virus (HIV), diarrhea in the last 2 weeks, cough in the last 2 weeks, stunting and wasting), environmental conditions (cooking place, fuels used for cooking, smokers in the house, family size).

A structured questionnaire was developed based on the literature available on the subject [21, 23–26], and a medical chart used to collect data for patients in the hospitals. Six data collectors and two supervisors were selected. Senior health officers and nurses who trained on Integrated Management of Neonate and Child Illness (IMNCI) were involved in the data collection. Training for one day was provided to the data collectors and supervisors on objectives of the study, steps, and approaches for interviewing, to take accurate and proper measurements of weight, height/length and calculate height for age (H/A), weight for height (W/H) and how to take correct vital signs and physical examinations. The data were collected in phases, the first data were taken on admission date as baseline information, and the second was on the third day about the clinical prognosis and antibiotics taken. It is concerned with the clinical status of the patient, either stable, same, or worsen. The final data were collected on the discharge time. If the child was not discharged, the final data were collected on the sixth to the eighth day, date of discharge, discharging conditions (improved same, none improved or death), antibiotics are taken, and treatment failure and other important information were registered. If the final diagnosis was other than severe community-acquired pneumonia, the case was excluded.

### Data processing and analysis

Data were collected using EPI DATA, version 3.02; and were exported to R statistical programming language version 4.0.5 for analysis. Descriptive statistics such as mean, standard deviation (SD), median, interquartile range (IQR), and percentages were performed and presented in tables.

Bivariate logistic regression analysis was performed to show unadjusted associations between each prognostic factor and poor outcomes of severe community-acquired pneumonia and to select potential candidate prognostic determinants for multivariate prediction modeling. Variables with a p-value less than 0.25 in the bivariate regression were retained for multivariate analysis. We performed multicollinearity tests between each determinant using variance inflation factor (VIF), we used VIF>10 as a cut-off point to exclude a variable for multivariate modeling [27].

A backward stepwise logistic regression analysis was performed to come up with the final reduced model. The regression coefficients with their 95% confidence levels and p-values were reported for the models. Model accuracy was checked using the area under the ROC curve (discrimination) and calibration plot (calibration) respectively. Interpretation of discrimination ability of a model; an AUC of 0.5 is worthless, and AUC from 0.80-.90 and 0.90–1.0 is good and excellent model performance respectively [28, 29].

To internally validate our model, a bootstrapping technique was used. For model derivation, we had drawn 1000 random bootstrap samples with replacement. Similar to the original model, the regression coefficients and AUC of the validated model were reported and compared with the original model. We used a decision curve analysis (DCA) to evaluate the clinical and public health impact of the model of standardized net benefit across a range of threshold probabilities (0 to 1) [30].

To develop a simplified and easily applicable prediction score for the outcomes of severe community-acquired pneumonia, each regression coefficient in the validated model was divided by the smallest coefficient and rounded to the nearest integer. We determined the total score for each individual by assigning the points for each variable present and adding them up. For simple interpretation in a clinical setting, we categorized the total risk score into two based on the Youden index (optimal cut-off point). Then, patients were categorized into high-risk or low-risk groups based on the summation of individualized risk scores. Since, a simplification of a risk score might cause loss of information, which might result in some loss in prognostic accuracy; we created a model for the risk score to compare its accuracy with the original one. So, ROC was plotted and an AUC with its 95% confidence level was computed to evaluate the discriminatory power of the scoring system.

### Ethics approval and consent to participate

Ethical clearance was obtained from the Ethical Review Committee of Bahir Dar University. Letters were written from Bahir Dar University to each study hospital for a corporation. Permission letters were obtained from each hospital administrative office to conduct the study on the ground. The study participant’s information sheet was attached to the front page of each questionnaire; before proceeding to the data collection process, the caregiver/family of each patient was asked for participation and well-informed. Verbal informed consent was received from the caregiver/family of the patient following explaining the purpose of the study.

## Results

### Baseline demographic and clinical characteristics of under-five with pneumonia

Regarding to the nature of our dataset, there is no missing data. We aimed for 565 children to be included in our study; however, 539 children were included in this study, giving a response rate of 95%. In this study, 270(50.1%) were females. The median age of the children was 17 months (IQR: 34–8), 200(37.1%) were in the ranges of 2 to 11 months age. One hundred forty two (26.3%), and 65 (12.1%) of children had cyanosis and paleness upon admission. This study also indicated that 239(44.3%) had vomiting, 243(45.1%) were unable to breastfeed, 138 (25.6%) had impaired consciousness, 107 (19.9%) were wasted, and 101(18.7%) were stunted, 83(15.4%) were admitted to ICU.The mean body temperature was 37.6 (±1.0 SD), and the median HCT was 33.2(IQR: 37.6–33.2) and. The median respiratory rate was 56(IQR: 62–48) (Table 1).

**Table 1:** Baseline demographic and clinical characteristics of under-five children with pneumonia admitted at hospitals in Amhara region, Ethiopia.

Variables	Category	Frequency	Percent (%)
Sex	Female	270	5.1
	Male	269	49.9
Age of child in month	2–11	200	37.1
	12–23	17	19.9
	24–35	18	20
	36–47	65	12.1
	48–59	59	10.9
Vaccination status	Fully vaccinated	287	53.2
	Incomplete	252	46.8
Diarrhea in the last 15 days	Yes	134	24.9
	No	45	75.1
Respiratory tract infection in the last 15 days	Yes	189	35.1
	No	35	64.9
Vomiting	Yes	239	44.3
	No	300	55.7
Cyanosis	Yes	142	26.3
	No	397	73.7
Pallor	Yes	65	12.1
	No	474	87.9
Unable to breastfeed	Yes	243	45.1
	No	296	54.9
Impaired consciousness	Yes	138	25.6
	No	401	74.4
Wasting	Yes	107	19.9
	No	432	8.1
Stunting	Yes	101	18.7
	No	438	81.3
Complete blood count finding	Positive	128	23.7
	Negative	411	76.3
Admitted to ICU	Yes	83	15.4
	No	456	84.6

### The incidence of poor outcomes and prognostic determinants of pneumonia among under-five children

The findings of this study revealed that the proportion of treatment failure, switching treatment, staying longer in the hospital, and death rates were 51(33.6%), 34(22.7%, 61(40.7%) and 5(3%) respectively. However, the overall incidence of poor outcomes of pneumonia was 151(28%) (95%CI: 24.2%-31.8%). We conducted a bivariate logistic regression analysis to identify prognostic determinants of poor outcomes of Pneumonia. Several prognostic determinants were entered in the bivariate modeling to select candidate predictors for the multivariate model. Accordingly, 14 candidate predictors were eligible for further regression analysis (Table 2).

**Table 2:** Bivariate regression analysis to develop and validate a prediction model for poor outcomes of Pneumonia in children.

Prognostic determinants	β(95%CI)	P-values
Sex(male)	0.32(-0.06–0.70)	0.098
Age of child months	-0.02(-0.03–0.01)	0.012
Vaccination status (fully vaccinated)	1.67(1.26–2.100)	<0.001
Fever(yes)	0.74(0.53–0.96)	<0.001
Cyanosis(yes)	2.62(2.17–3.08)	<0.001
Pallor(yes)	3.71(2.92–4.69	<0.001
Unable to breast feed	1.46(1.062–1.87)	<0.001
Impaired consciousness(yes)	2.84(2.37–3.32)	<0.001
Wasting(yes)	1.88(1.43–2.35)	<0.001
Stunting(yes)	0.92(0.47–1.37)	<0.001
CBC positive(yes)	1.55(1.13–1.97)	<0.001
Diarrhea (yes)	1.29(0.88–1.71)	<0.001
Admitted to ICU(yes)	3.85(3.13–4.69)	<0.001
Vomiting(yes)	1.67(1.28–2.11)	<0.001

### A predictive model for poor outcomes of severe community-acquired pneumonia in children

We entered 14 candidate prognostic determinants from the bivariate model, into original multivariate model. Prognostic determinants such as vaccination status, fever, pallor, unable to breastfeed, impaired consciousness, CBC positive, entered ICU, and vomiting remained in the reduced mode. To this end, we developed a prediction model, and risk scores based on the results of the reduced model (Table 3).

**Table 3:** β Coefficients and risk-scores of prognostic determinates to predict poor outcomes of pneumonia in children.

Prognostic determinants	Multivariate analysis			
	Original β		Bootstrap β	
	β(95%CI)	P-values	β(95%CI)	Risk score
Sex(M/F)	NA			
Age of child	NA			
Vaccination status (fully vaccinated)	0.69 (0.03–1.37)	0.039 *	0.69(-0.118–1.332)	2
Fever	0.38(0.04–0.72)	0.028 *	0.37(-0.039–0.794)	1
Cyanosis(yes)	NA			
Pallor(yes)	2.27 (1.06–3.59)	<0.001**	2.27(0.879–3.546)	6
HCT	NA			
Unable to breast feed (yes)	0.67 (0.05–1.31)	0.034*	0.68(-0.079–1.307)	2
Impaired consciousness	1.84 (1.16–2.53)	<0.001***	1.84(1.064–2.452)	5
Wasting (yes)	NA			
Stunting (yes)	NA			
CBC positive	0.79(0.01–1.57)	0.043*	0.79(0.043–1.545)	2
Diarrhea in the last 2 weeks (yes)	NA			
Admitted to ICU (yes)	2.04 (0.86–3.35)	0.001* *	2.04(0.690–3.346)	6
Vomiting (yes)	1.41 (0.78–2.07)	<0.001***	1.14(0.692–1.968)	3

**Fig 1:**
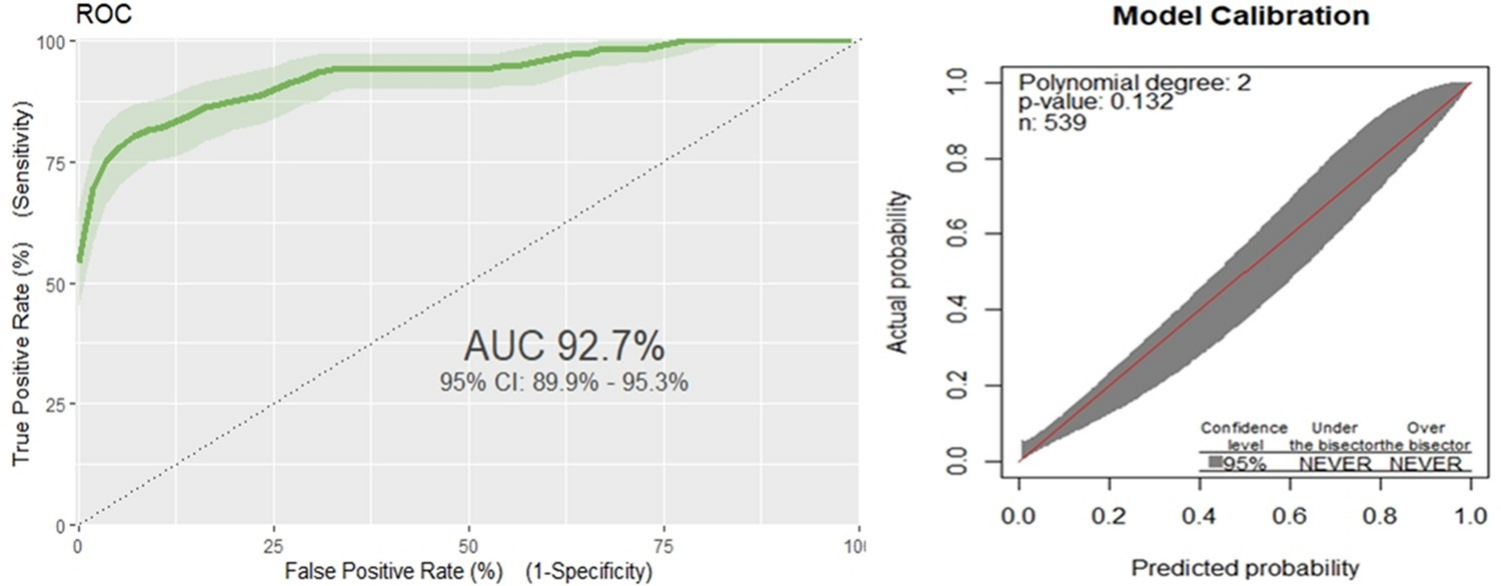
Receiver operating characteristics curve for prediction model of the original model (a), Calibration belt for the original model: predicted probability versus observed probability poor outcomes of SCAP in under-five children (b).

The area under the receiver operating characteristics curve (AUC) of the original model was 0.926(95% confidence interval 0.897 to 0.952) (Fig 1A). The model fitness test had a p-value of 0.176; the calibration curve is nearly 45 degrees, showing that there is no difference between predicted and the observed probabilities (Fig 1B).

Based on the default 0.5 cut off probability, the original model has accuracy (ACC) of 0.899 (10% misclassification rate), sensitivity(S) 0.742, specificity (SP) 0.961, positive predictive value (PV+) 0.882, and negative predictive value (PV-) 0.905. However, based on the optimal cut of point (Youden index) cut off point (**S1 Fig**) 0.339 probability, the model has accuracy (ACC) of 0.898, sensitivity(S) 0.801,95%CI (0.729, 0.862), specificity (SP) 0.935,95%CI(0.906, 0.958), positive predictive value (PV+) 0.828,95%CI (0.763, 0.882), and negative predictive value (PV-) 0.923, 95%CI (0.889, 0.950). The density plot of the original multivariate model indicated that 28% of the study subjects were with poor outcomes of SCAP (positive cases). The graph with red one represents children with low risk of SCAP, and the blue one was children at high risk of poor outcomes of SCAP. The plot showed some overlap in the model, revealing, it isn’t 100% perfect (**S2 Fig**).

**Fig 2:**
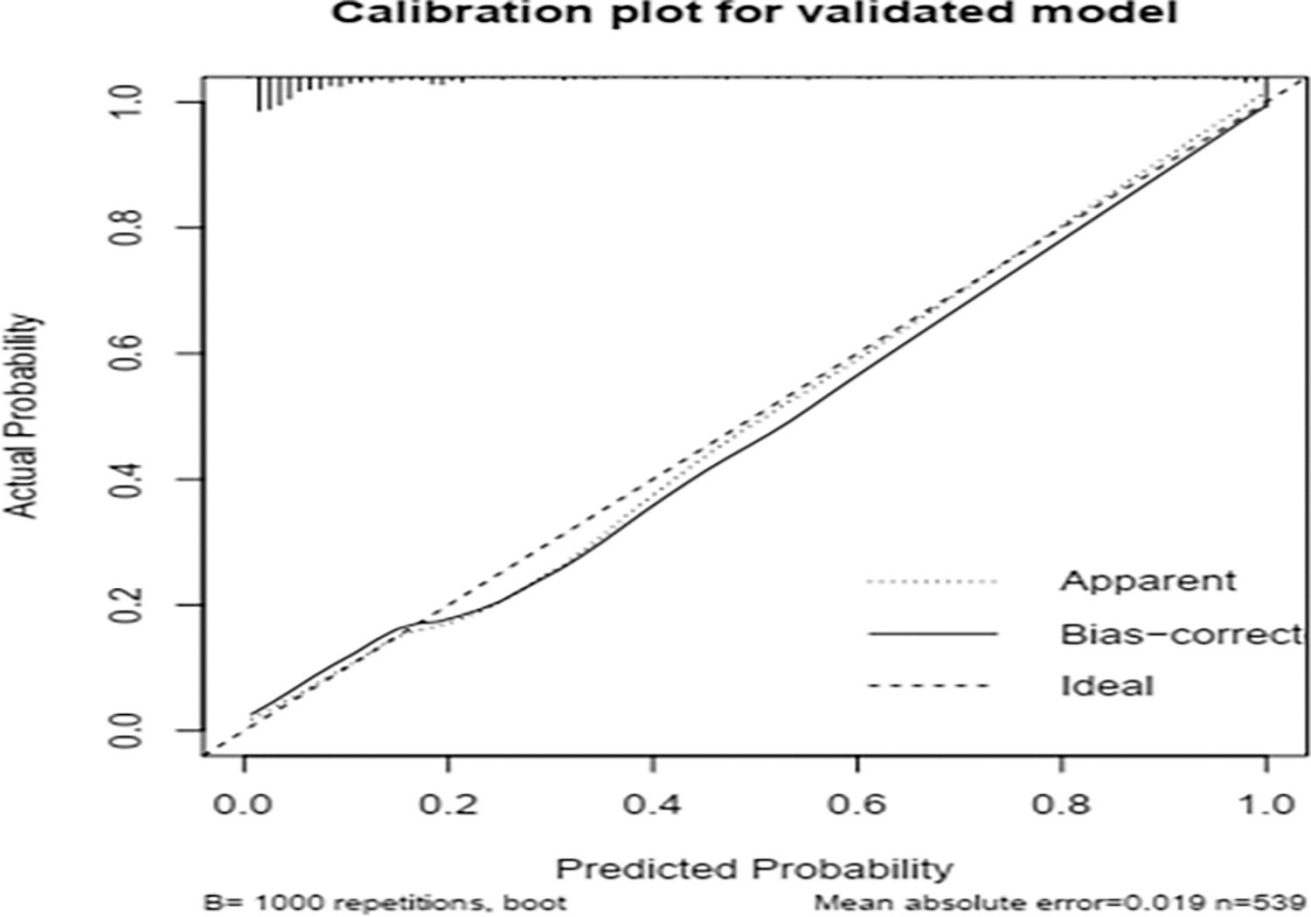
Calibration plot of the validated model: Predicted probability versus actual probability of poor outcomes of SCAP in under five children.

To avoid over-interpretation and minimize too optimistic results from the original model; we used a bootstrapping technique using mrs package to validate our model. This study used 1000 bootstrap samples with replacement; the corrected AUC was 0.913, 95%CI (0.899, 0.956) and the optimism coefficient for the validated model was 0.0135. The β coefficients from the bootstrapped model produced marginally the same results as the original β coefficients. The calibration plot for the validated model is shown (Fig 2); indicates a very good agreement between predicted and observed probabilities; very slightly that the apparent curve seems to outperform the bias-corrected curve between 0.2 and 0.7 predicted probabilities. Therefore, given the limited optimism, and excellent calibration, the model might perform well in a new sample.

To predict an individual estimated risk of poor outcomes of pneumonia based on linear predictors of validated regression coefficients as: the probability of poor outcomes of pneumonia = 1/1+exp-(-15.64+0.69*fully vaccinated + 0.37*fever+ 2.27*pallor (yes) + 0.68*unable to breastfeed+ 1.84*impaired consciousness + 0.79*CBC positive + 2.04*admitted to ICU + 1.14*Vomiting (yes).

**Fig 3:**
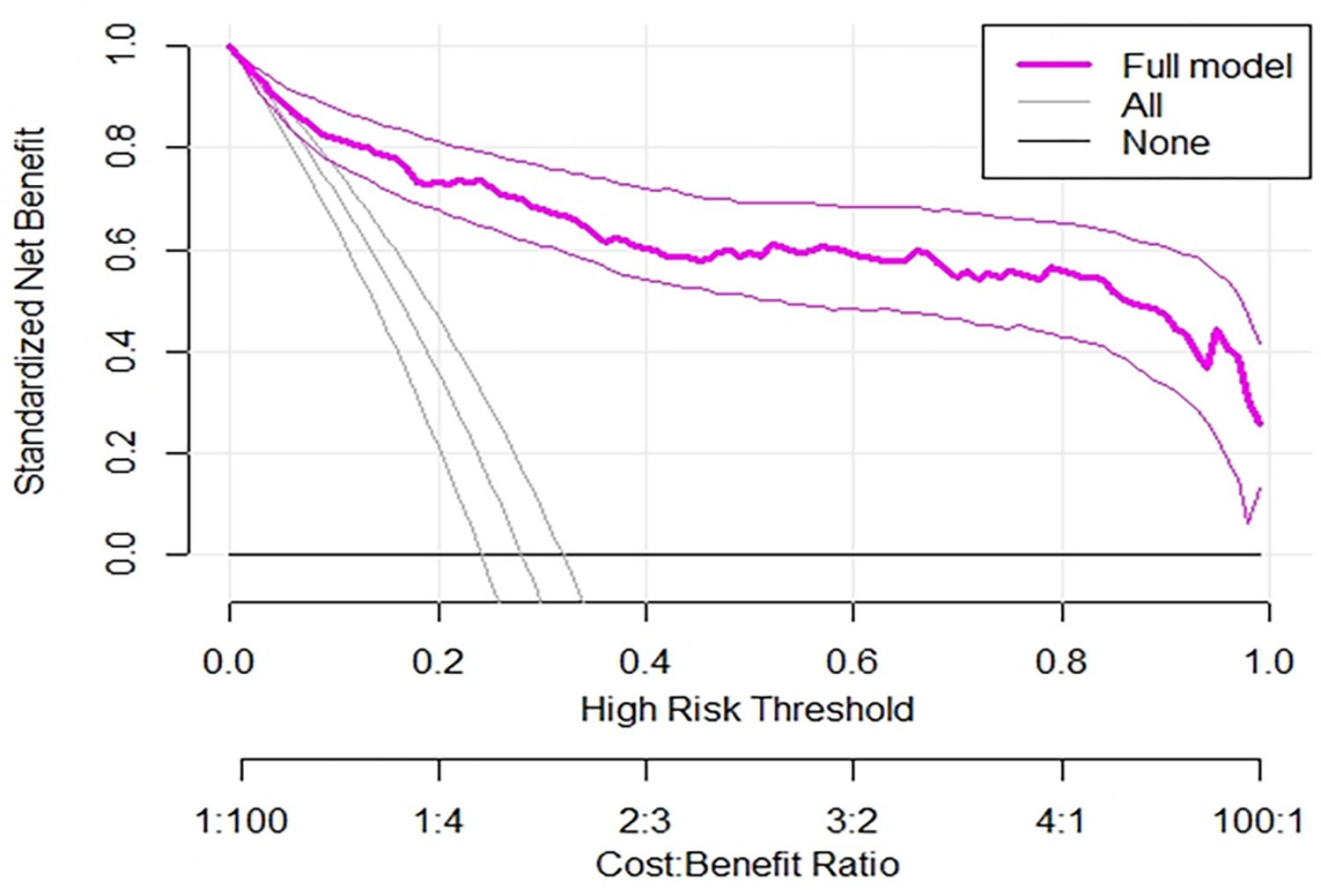
A plot of decision curve illustrating the net benefit of the model against threshold probability and corresponding cost-benefit ratio of poor outcomes of SCAP in under-five children.

Regarding the decision to use our model, the decision curve outperforms the default strategies (referring all and none) across the entire range of threshold probabilities. This implies that our model has the highest clinical and public health importance. Therefore, decisions made using the model such as safely discharging children with some medications or keeping children for more intensive care in the hospitals has a higher net benefit (Fig 3).

### Risk classification using a simplified risk score

**Fig 4:**
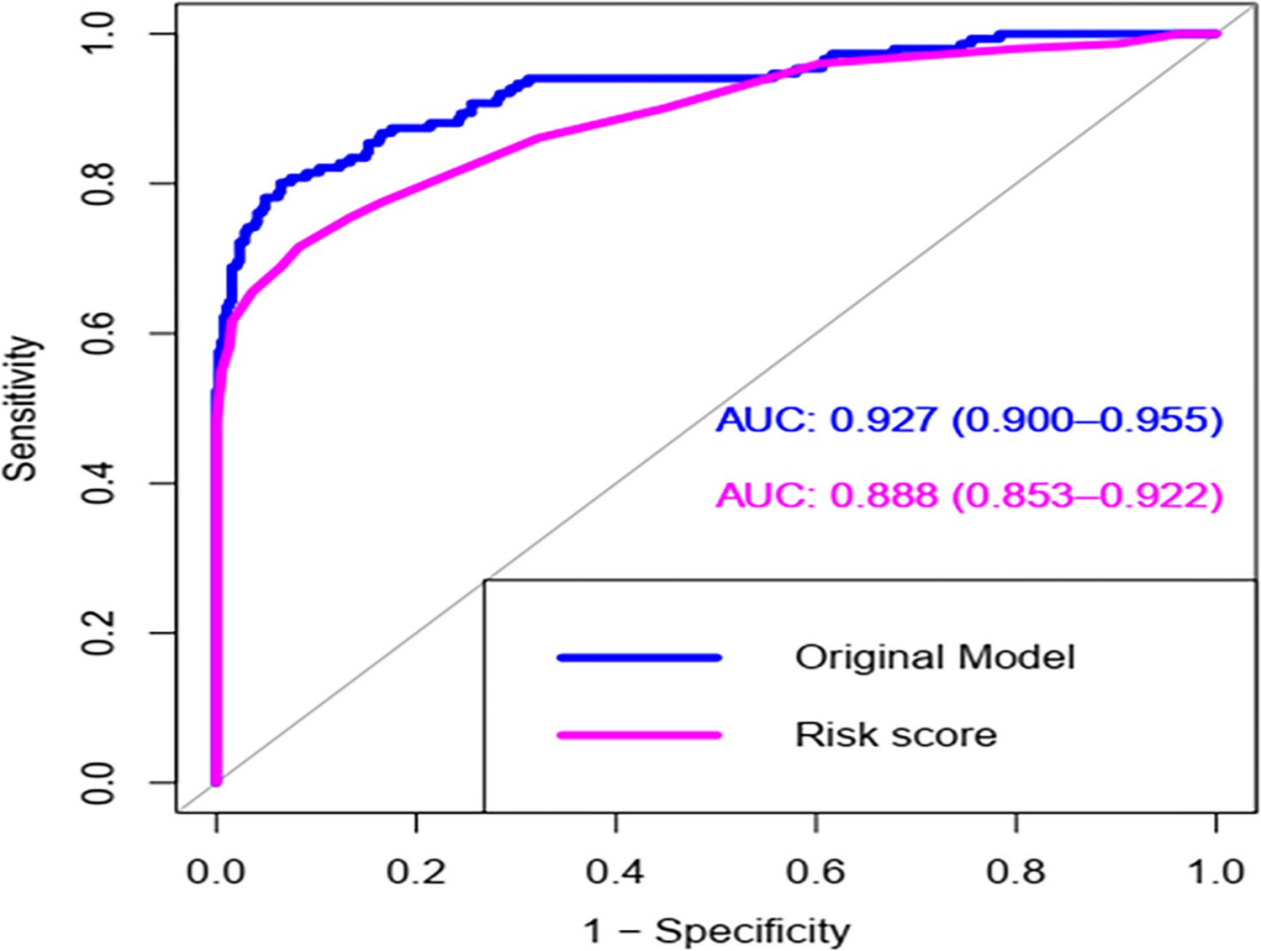
ROC curve for original β coefficient (blue curve) versus ROC curve for simplified risk score model (reddish-purple curve) for poor outcomes of SCAP in under-five children.

For practical utility, we developed a simplified risk score from the validated model. The risk score produced prediction accuracy of an AUC of 0.89 (95%CI: 0.853–0.922); which is nearly a comparable prediction accuracy with the original model, a curve with reddish-purple color (See Fig 4). This reveals that the probability of a randomly selected child with poor outcomes of pneumonia will receive a higher risk score than a randomly selected child without poor outcomes of pneumonia is 89%.

In this study the maximum total risk score is 27; for simple interpretation in the clinical settings, we categorized risk scores into less than nine points (< 9) (low-risk group), and greater or equal to nine points (≥ 9) points (high-risk group) based on Youden index (optimal cut-off point) which corresponds to the probability of 0.333 in the model. Therefore, a child can have a minimum and maximum risk score of 0 and 27 respectively. Out of the total 151(28%) cases of poor outcomes of SCAP, 47(11.5%) were in the low-risk group, and 104(81%) were in the high-risk group (Table 4).

**Table 4:** Risk stratification for poor outcomes of pneumonia using simplified prediction score.

Risk category	Frequency	Incidence of poor outcome
Low (<9 score)	410	47 (11.5%)
High (> = 9 score)	129	104 (81%)
Total	539	151(28%)

Score = (vaccination full*2) + (fever*1)+(Pallor*6)+ (Unable to breast feed*2)+(impaired consciousness*5)+(CBC positive*2)+(admitted to ICU*6)+(vomiting*3)

As to the risk score model performance, it had accuracy of 0.861, 95%CI (0.828, 0.889); indicating that the misclassification rate for the risk score model is less than 14%. The model has a Sensitivity 0.715, 95%CI (0.636, 0.786), specificity 0.918, 95%CI (0.886, 0.943), PPV 0.771, 95%CI (0.701, 0.831), NPV 0.892, 95%CI (0.852, 0.925).

## Discussions

To halt the loss of millions of young lives from preventable causes including pneumonia, the World Health Organization and UNICEF set an integrated Global Action Plan for the Prevention and Control of Pneumonia and Diarrhea (GAPPD). The plan had targeted less than three child pneumonia deaths per 1,000 live births by 2025. The plan brings together critical services and interventions to create healthy environments, encourages activities that save children from diseases in order to ensure every child has the access to recognized and appropriate preventive and clinical interventions [31]. Similarly, the Sustainable Development Goal (SDG) targeted less than 25 under-five child death per 1,000 live births by 2030 [32].

As part of these targets, accurate risk-stratification tools and models to guide clinical decision-making in the health care settings are very essential. Therefore, we developed a prediction model and risk scores for poor outcomes of SCAP for under-five children. A total of 539 under-five children admitted with SCAP in the hospitals were studied, and the incidence of poor outcomes of SCAP was 28%. We predicted poor outcomes of SCAP by using prognostic determinants that remained in our reduced model (vaccination status, fever, Pallor, unable to breastfeed, impaired consciousness, vomiting on admission, CBC positive, and being admitted to ICU).

The findings of this study revealed that our model has produced discrimination performance of AUC 0.927(95% confidence interval 0.90 to 0.96) with calibration a p-value of 0.132; the calibration curve is almost in 45 degrees, showing a magnificent agreement between predicted and the observed probabilities (Fig 1B). To make internal validation of our study, a bootstrapping technique was used to minimize too optimistic results in the original model. We used 1000 bootstrap samples with replacement; the adjusted AUC was 0.913, 95%CI (0.899, 0.956) and optimism for the validated model was 0.0135. The bootstrapped model has produced marginally the same β coefficients as the original model; and the calibration plot for the validated model is shown (Fig 2); indicates fits well over the ranges of predicted probabilities. Therefore, given the limited optimism, and excellent calibration, the model might perform well in a new sample in the future. To this end, our risk score model has magnificent discrimination ability, with an AUC of 0.89 (95%CI: 0.85–0.92).

To our knowledge, there are no prediction models for poor outcomes of SCAP for children presented with severe community-acquired pneumonia in children less than five in low and middle-income countries; however, several prognostic models were available to predict mortality in children hospitalized with SCAP.

A study conducted by Dana W Flanders and his colleagues, the area under the ROC curve for the Pneumonia Severity Index (PSI) model to predict in-hospital mortality was 0.847, with a calibration p-value of < 0.001 [26]. A prediction study on mortality in children with Respiratory Illness in western Kenya using a modified Respiratory Index of Severity in Children (RISC); the model resulted in an AUC of 0.854 with optimism coefficient of 0.002 [33]. Similarly, an area under the ROC curve of another Pneumonia Severity Index (PSI) study to predict mortality was 0.84 with a p-value < 0.0001 [34]. The findings of these studies showed that the discrimination performance of tools to identify children at greater risk of death from SCAP is very good. The accuracy of our model is slightly higher than a study conducted in Gambia, where the aim of this study was to predict the mortality due to SCAP in children, and the model had an AUC of 0.88 (95% confidence interval: 0.84, 0.91), sensitivity was 0.78 and specificity was 0.77 [35].

However, our model performance is much higher than a study conducted in Malawi to predict in hospital mortality in children from pneumonia, two models were used for external validations; the Respiratory Index of Severity in Children (RISC) and the modified RISC (mRISC) scores in child pneumonia. The models produced discrimination performance of 0.72, and 0.79 respectively [36]. Showing that, the models have produced good discrimination ability among children with pneumonia.

In a study conducted in the US to predict severe outcomes of SCAP, the model had an accuracy of AUC 0.79 (0.77–0.81). This model is slightly different from ours as it strived to predict severe outcomes of SCAP using mechanical ventilation, shock, or death, while ours is aimed at poor outcomes of SCAP following admission using treatment failure, antibiotic change, prolonged hospital stay, and death during hospitalization. In addition, the earlier study used radiologic infiltrate patterns, and microbiologic data in addition to physical and clinical presentations [37].

Biomarker studies conducted to assist clinical decision-making on community-acquired pneumonia were revealed that inflammatory biomarkers including C-reactive protein (CRP), procalcitonin (PCT), cytokines [38–40], and cardiovascular biomarkers such as N-terminal B-type natriuretic peptide (NT-proBNP), proarginin-vasopressin (copeptin), and D-dimer can predict mortality and disease severity in CAP [41–44]. However, these mechanisms are less likely to be implemented and to be used in routine patient care practice in low and middle-income countries.

Most of the available prediction tools and risk scores for SCAP were conducted to predict mortality and identify low-risk patients that are suitable for ambulatory management and admission to the hospitals. However, identification of patients at high risk for poor outcomes by the existing scores remains suboptimal. Hence, it is essential to conduct a repeated evaluation of hospitalized patients to account for the risk of poor outcomes in the course of the illness. So, our prognostic determinants that predicted SCAP were vaccination status, fever, Pallor, unable to breastfeed, impaired consciousness, vomiting on admission, CBC positive, and being admitted to ICU. These variables are easy to be collected on a bedside that might be used to predict the risk of poor outcomes of a given individual patient for a prompt decision to complement the clinical judgment by health care workers.

### Strength and limitations

We developed a risk stratification tool by using easily obtainable prognostic determinants to assist clinical decision making that can be utilized in low and middle income settings, where the availability of imaging and lab tests are limited. However, our findings should be used with great caution because this study should be externally validated before use. The prognostic outcome isn’t adjusted for interventions provided.

## Conclusions

We developed well-calibrated predicted models that have an excellent discrimination performance for poor outcomes of SCAP among children admitted to the hospitals. Our model has 8 prognostic determinants that can be easily collected from each patient following admission. These variables are easy to be obtained from the diagnostic workup to predict the outcomes of the patients with SCAP. Therefore, our risk score tool could be used in the clinical care settings to identify children at low risk for poor outcomes that could be safely discharged with some medications, and those with a high risk of poor outcomes that require intensive management. External validation is a prerequisite and highly recommended before the implementation of our prediction tool. After external validation, the implementation of our prediction score model can facilitate patient management decisions by offering individualized risk estimates that can be utilized with clinical judgment to enhance the recovery of children with SCAP.

## Supporting information

S1 Checklist TRIPOD checklist: Prediction model development. (DOCX)

S1 Fig Optimal cut off value in ROC curve (Youden index) for the original model. The black dot on the curve represents cut off the predicted probability (0.339), and sensitivity and specificity values in bracket.
(TIF)

S2 Fig Density plot of original model (children with low risk of poor outcomes of SCAP, red in color versus children with high risk of poor outcomes SCAP blue in color). (TIF)

S1 Dataset Full dataset for validation of risk prediction for outcomes of severe community-acquired pneumonia among under-five children in Amhara region, Northwest Ethiopia. (SAV)
