# Supplementary figures and images for: Validation of risk prediction for outcomes of severe community-acquired pneumonia among under-five children in Amhara region, Northwest Ethiopia
Source: PLoS One. 2023 Feb 15;18(2):e0281209. doi: 10.1371/journal.pone.0281209 (PMC9931104; doi:10.1371/journal.pone.0281209)

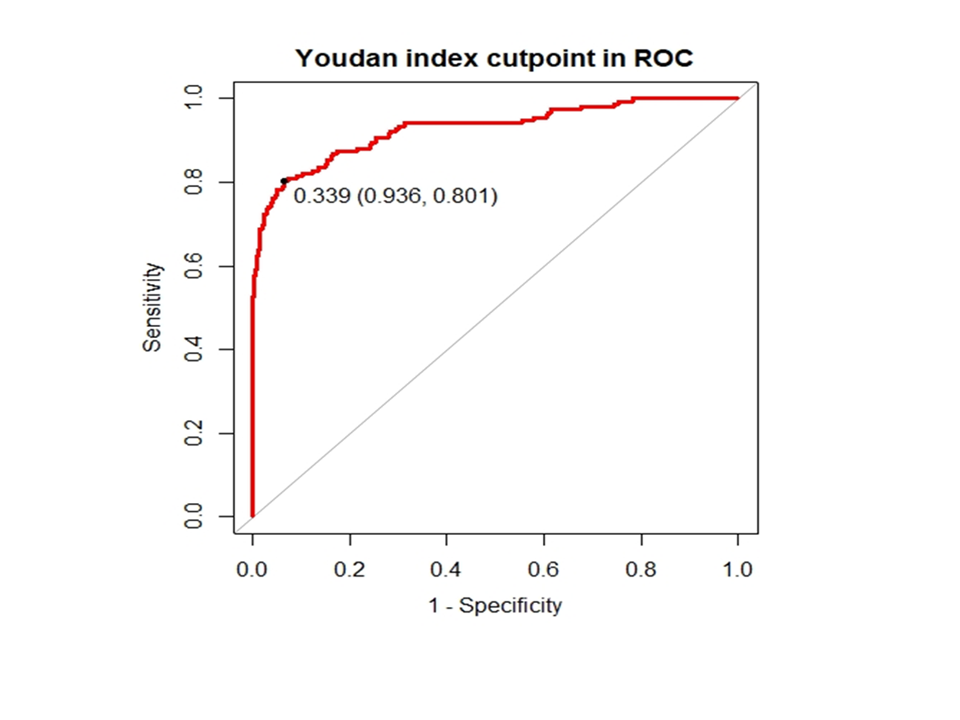

Supplement: S1 Fig — The black dot on the curve represents cut off the predicted probability (0.339), and sensitivity and specificity values in bracket. (TIF) [file pone.0281209.s002.tif]

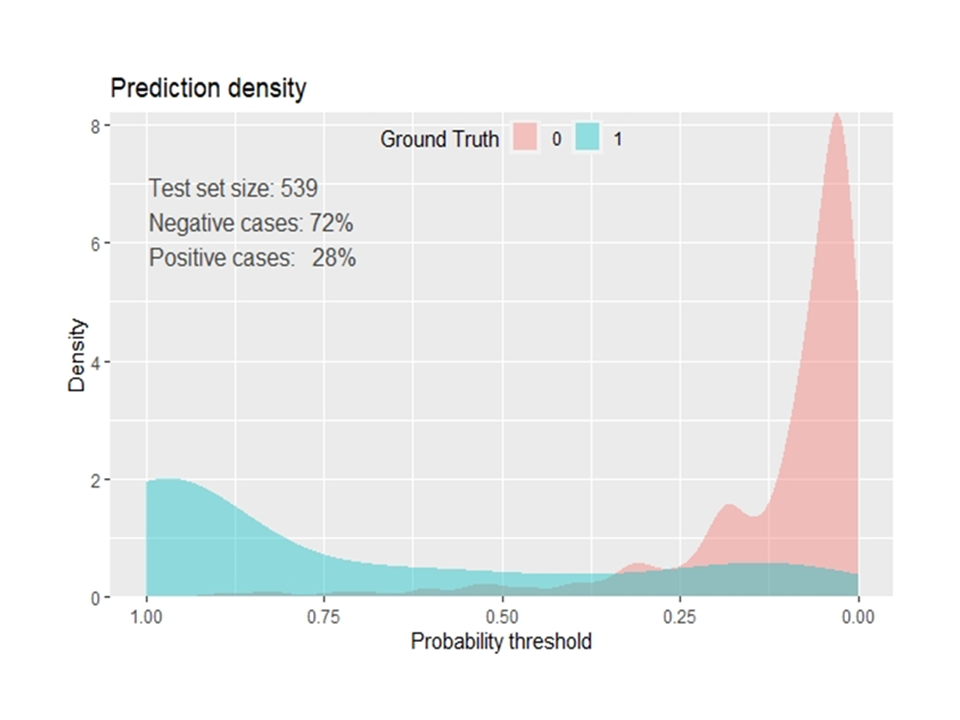

Supplement: S2 Fig — (TIF) [file pone.0281209.s003.tif]
